# Safety evaluation on low-molecular-weight hydroxyethyl starch for volume expansion therapy in pediatric patients: a meta-analysis of randomized controlled trials

**DOI:** 10.1186/s13054-015-0815-y

**Published:** 2015-03-10

**Authors:** Lixia Li, Yongyang Li, Xiaoxing Xu, Bo Xu, Rongrong Ren, Yan Liu, Jian Zhang, Bin He

**Affiliations:** Department of Pharmacy, Xinhua Hospital, Shanghai Jiaotong University School of Medicine, Kongjiang Road 1665, Shanghai, 200092 China; Department of Surgery, Shanghai Jiaotong University Affiliated Sixth People’s Hospital, Yishan Road 600, Shanghai, 200233 China; Department of Epidemiology, Shanghai Jiaotong University School of Medicine, Kongjiang Road 1665, Shanghai, 200092 China; Department of Anesthesiology and SICU, Xinhua Hospital, Shanghai Jiaotong University School of Medicine, Kongjiang Road 1665, Shanghai, 200092 China

## Abstract

**Introduction:**

Hydroxyethyl starch (HES) has been widely used for volume expansion, but its safety in adult patients has been questioned recently. The aim of this meta-analysis is to see whether or not HES has any adverse effect in pediatric patients.

**Methods:**

Randomized controlled trials (RCTs) involving pediatric patients who received 6% low-molecular-weight HES, published before January 2014, were searched for in Pubmed, Embase database and Cochrane Library. Two reviewers independently extracted the valid data, including the mortality, renal function, coagulation, blood loss, hemodynamic changes, and length of hospital and ICU stay. All data were analyzed by *I*^2^-test, and the results of statistical analysis were displayed in forest plots. Possible publication bias was tested by funnel plots. Bayesian analysis was performed using WinBUGS with fixed and random effects models.

**Results:**

A total of 13 RCTs involving 1,156 pediatric patients were finally included in this meta-analysis. Compared with other fluids, HES did not significantly decrease the mortality (RR = -0.01; 95%CI: 0.05 to 0.03; *P* = 0.54; *I*^2^ = 6%), creatinine level (*I*^*2*^-test: MD = 1.81; 95%CI: -0.35 to 3.98; *P* = 0.10;*I*^*2*^ = 0%; Bayesian analysis: Fixed effect model MD = 1.77; 95%CI: -0.07 to 3.6; Random effects model MD = 1.78; 95%CI: -1.86 to 5.33), activated partial thromboplastin time (MD = 0.01; 95%CI: -1.05 to 1.07; *P* = 0.99; *I*^2^ = 42%), and blood loss (MD = 17.72; 95%CI: -41.27 to 5.82; *P* = 0.10; *I*^2^ = 0%) in pediatric patients. However, HES significantly decreased the blood platelet count (MD = 20.99; 95%CI: -32.08 to -9.90; *P* = 0.0002; *I*^2^ = 28%) and increased the length of ICU stay (MD = 0.94; 95%CI: 0.18 to 1.70; *P* = 0.02; *I*^2^ = 46%).

**Conclusions:**

Volume expansion with 6% HES significantly decreased the platelet count and increased the length of ICU stay, also might have an adverse effect on renal function. Therefore HES is not recommended for pediatric patients, which safety needs more high quality RCTs and studies to confirm in future.

**Electronic supplementary material:**

The online version of this article (doi:10.1186/s13054-015-0815-y) contains supplementary material, which is available to authorized users.

## Introduction

Hydroxyethyl starch (HES) has been widely used for clinical volume expansion since the 1960s. However, a series of recent randomized controlled trials (RCTs) [1-3] have questioned the safety of HES for volume expansion in adult patients, suggesting that HES may increase the mortality and the incidence of renal replacement therapy. Based on these studies, the European Society of Intensive Care Medicine (ESICM) recommends that HES should not be used for volume expansion due to the high risk for inducing kidney injury and bleeding [4]. However, this recommendation is mainly based on the findings in adult patients. Whether HES has similar adverse effects in pediatric patients and whether it can be used safely in children remain unanswered, knowing that children have different pulmonary, vascular and cardiac compliances, and different responses to volume expansion therapy as compared with adult patients [5-7]. The objective of this meta-analysis was to evaluate the safety of HES versus other fluids for plasma volume expansion in pediatric patients.

## Materials and methods

No ethical approval was required because this study includes no confidential personal data or interventions on the patients according to the Preferred Reporting Items for Systematic Reviews and Meta-Analyses (PRISMA) [8]. Bayesian analysis on creatinine (Cr) was performed using WinBUGS (version 1.4.3, MRC Biostatistics Unit, Cambridge, UK) with fixed and random effects models developed by Dias [9]. We used 100,000 iterations after an initial burn-in of 1,000. This meta-analysis included RCTs for pediatric patients who received 6% low-molecular-weight HES. The primary parameters were the overall mortality, renal function, bleeding and coagulation function. The secondary parameters were hemodynamic parameters, the amount of fluid used for resuscitation, and the length of hospital and ICU stay. The inclusion criteria were: 1) RCTs; 2) patients ≤18 years old; and 3) studies that included a group of patients receiving 6% low-molecular-weight (130 kD and 200 kD) HES and a control group receiving other fluids. RCTs that met one of the following criteria were excluded: 1) no group receiving 6% low-molecular-weight HES; 2) no valid data available; and 3) studies from Dr. Joachim Boldt.

### Search strategies

We searched the PubMed and Embase databases and the Cochrane Library using the following key words and related free words: ‘hydroxyethyl starch’, ‘HES’, ‘child’, ‘children’ and ‘pediatric’. The relevant clinical trials were those published before 19 January 2014 that met the above criteria. The search was limited to ‘randomized controlled trials’, ‘human’ and ‘children’, and the language was restricted to English. The details are shown in Additional file 1.

### Study selection and data extraction

Two reviewers (LL and YL) independently screened the search results and obtained the full texts according to the inclusion and exclusion criteria, and independently extracted the valid data. Data extraction and analysis were performed under the supervision of an experienced statistician (XX).

### Quality assessment

The literature quality was assessed by the Jadad scoring system [10]. A study with an overall score ≤2 was considered poor in quality and >2 was considered high in quality. We performed risk of bias assessment using the ‘Risk of bias’ tool in the Cochrane Handbook for Systematic Reviews of Interventions [11]. We assessed each study according to the quality domains of random sequence generation, allocation concealment, blinding of participants and personnel, incomplete outcome data, selective outcome reporting and other bias.

### Statistical analysis

Data were analyzed by Review Manager (5.2 RevMan, Cochrane Collaboration). The pooling continuous effect data were assessed by mean difference (MD). When median and extreme values were presented in the original articles, these data were converted into mean and standard deviation according to relevant formulas [12]. Pooling non-continuous data were assessed by the risk ratio (RR). If there was more than one group, data were pooled as one group. Statistical heterogeneity of the data was analyzed quantitatively by the *I*^2^-test [13]. The fixed effects model was selected if no heterogeneity existed (*I*^2^ < 50%), and the random effects model was selected in the event of 50% ≤ *I*^2^ < 75%. A sensitivity analysis or subgroup analysis would be performed to exclude the heterogeneity if *I*^2^ ≥ 75%; otherwise only descriptive analysis would be performed without meta-analysis. Publication bias was tested by funnel plots. Two-sided tests were performed with a significant difference at *P* <0.05.

## Results

### Literature search and study selection

**Figure 1:**
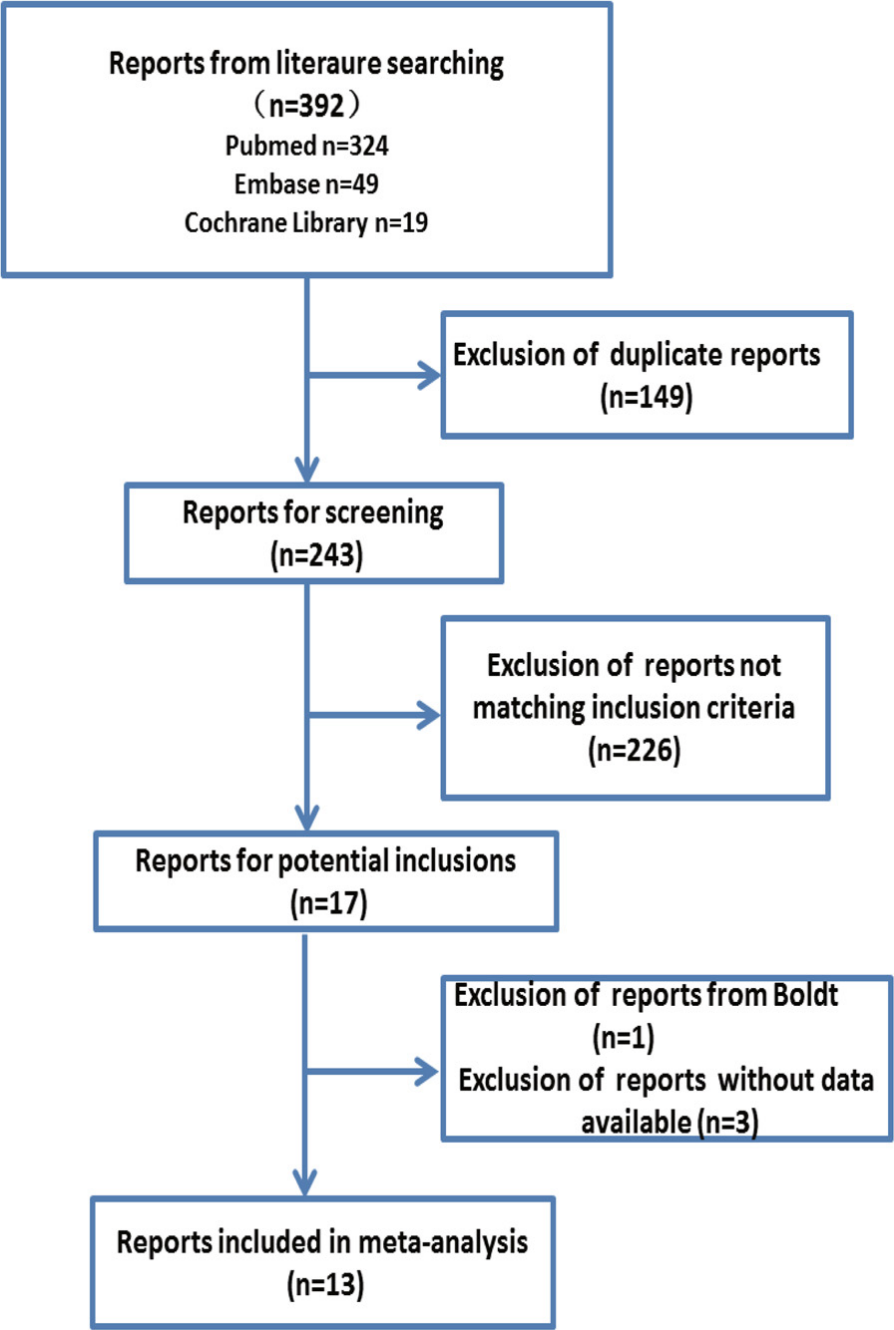
**The flow chart of the literature search.**

Figure 1 is the flow chart of the literature search, which identified a total of 392 articles, from which 379 were excluded after reading the titles, abstracts and full texts. Finally, 13 RCTs were included in this meta-analysis.

### Characteristics of the articles

The characteristics of the 13 RCTs, involving 1,156 patients, are shown in Table 1. Four RCTs [14-17] reported the mortality; five RCTs [14,17-20] reported the effect on renal function; nine RCTs [15,17-19,21-25] reported the effects on bleeding and coagulation; seven RCTs [14-17,19,25,26] reported hemodynamic changes; six RCTs [15,17,19-21,25] reported the amount of fluid replacement; four RCTs [15,17,19,20] reported the length of ICU stay; and four RCTs [15,19,20,23] reported the total length of hospital stay.

**Table 1:** **Characteristics of the included randomized controlled trials**

**Trial**	**Indication**	**HES group**				**Control group**			
		**Program**	**Patients (n)**	**Age**	**Weight (kg)**	**Program**	**Patients (n)**	**Age**	**Weight (kg)**
Liet *et al*. 2006 [14]	Hypotensive neonates with low cardiac output and absence of myocardial dysfunction	6%HES 200/0.5	7	5 ± 6d	1.4 ± 0.7	5% Albumin Isotonic saline	7	2 ± 1d	1.3 ± 0.6
							7	6 ± 10d	0.9 ± 0.3
Standl *et al*. 2008 [15]	Non-cardiac surgery	6%HES 130/0.4	41	8.3 ± 9.2 m	8.0 ± 6.6	5% Albumin	40	8.7 ± 10.9 m	7.1 ± 4.6
Akech *et al.* 2010 [16]	Severe falciparum malaria	6%HES 130/0.4	40	40.3 ± 12.8 m	-	6% Dextran	39	40.3 ± 12.8 m	-
Van Der Linden *et al*. 2013 [17]	Undergoing elective surgery for congenital heart disease	6%HES 130/0.4	31	6.1 ± 5.2Y	25.1 ± 24.6	5% Albumin	29	4.8 ± 3.7y	16.7 ± 10.3
Liet *et al*. 2003 [18]	Plasma volume expansion with HES in the newborn	6% HES 200/0.5	13	3 ± 2.2d	1.27 ± 0.47	5% Albumin	13	5 ± 3d	1.33 ± 0.64
Hanart *et al*. 2009 [19]	Undergoing cardiac surgery with cardiopulmonary bypass	6%HES 130/0.4	60	23.5 ± 19.8 m	9 ± 4	4% Albumin	59	17.3 ± 20.0 m	8 ± 4.3
Akkucuk *et al*. 2013 [20]	Children undergoing cardiac surgery	6%HES 130/0.4	12	3.9 ± 1.7y	13.5 ± 4.5	Ringer’s acetate	12	5.1 ± 3.7y	16.2 ± 8.8
Chong Sung *et al*. 2006 [21]	Undergoing elective repair of atrial septal defect, ventricular septal defect or tetralogy of Fallo	6%HES 130 ⁄0.4	21	31.5 ± 34.6 m	13.7 ± 8.9	Fresh frozen plasma	21	43.5 ± 61.6 m	15.7 ± 13.4
Haas *et al*. 2007 [22]	Prevention of intra-operative hypovolemia	6% HES 130 ⁄0.4	14	9 ± m	9 ± 2	5% Albumin 4% Gelatine	14	14 ± 9 m	10 ± 2
							14	10 ± 10 m	8 ± 3
Wills *et al*. 2005 [23]	Dengue shock syndrome	6%HES 200/0.5	129	9.6 ± 4.9y	26 ± 13.3	5% Dextran Ringer’s lactate	126	10 ± 4.1y	27 ± 14.5
							128	9.8 ± 4.6y	26.8 ± 13.9
Wills *et al*. 2005 [23]	Dengue shock syndrome	6%HES 200/0.5	62	9.3 ± 4.6y	24.8 ± 14.1	5% Dextran	67	8.8 ± 4.6y	25.5 ± 13.4
Sahoo *et al*. 2007 [24]	with CCHD undergoing modified BT shunt operations	6%HES 200/0.5	25	17.7 ± 12.3 m	7.0 ± 2.4	5% Dextran	25	19.5 ± 14.3 m	7.9 ± 2.4
Osthaus *et al*. 2009 [25]	Aged 0 to 12 years scheduled for surgery	6%HES 130/0.4	25	4.4 ± 6.4y	18.7 ± 22.2	4% Gelatin	25	4.6 ± 6.3y	20.2 ± 25.0
Witt *et al*. 2008 [26]	Major pediatric surgery	6% HES	25	33.2 ± 40 m	13.4 ± 11	4%Gelatin	25	38.7 ± 39 m	14.2 ± 10
		130/0.4							

### Quality of the included studies

As shown in Table 2, the study quality was assessed using the Jadad scoring system. Seven RCTs were of low quality (≤2) and six RCTs were of high quality (>2). More details about the risk of bias assessment are shown in Table 3. The overall risk of bias in four RCTs was low, and that in the other RCTs was unclear.

**Table 2:** **Assessment of literature quality**

**Literature resources**	**Randomization**	**Random sequence generation**	**Allocation concealment**	**Blinding methodology**	**Withdrawal and dropouts**	**Jadad score**
Liet *et al*. 2006 [14]	Yes	Computer-generated	Yes	Yes	6/0	5
Standl *et al*. 2008 [15]	Yes	Sealed randomization envelopes	Yes	None	13/1	4
Akech *et al*. 2010 [16]	Yes	Randomization cards	Yes	Unclear	16/0	4
Van Der Linden *et al*. 2013 [17]	Yes	By means of a macro written in SAS®, version 9.3.1.	Yes	Double-blinded	0/0	5
Liet *et al*. 2003 [18]	Yes	Unclear	Unclear	Double-blinded	Unclear	2
Hanart *et al*. 2009 [19]	Yes	The randomization assignment was concealed in an envelope	Yes	Double-blinded	Unclear	4
Akkucuk *et al*. 2013 [20]	Yes	Unclear	Unclear	Unclear	Unclear	1
Chong Sung *et al*. 2006 [21]	Yes	Unclear	Unclear	Unclear	Unclear	1
Haas *et al*. 2007 [22]	Yes	Unclear	Unclear	Unclear	Unclear	1
Wills *et al*. 2005 [23]	Yes	Computer-generated	Yes	Yes	0/1	5
Sahoo *et al*. 2007 [24]	Yes	Unclear	Unclear	Unclear	0/7	2
Osthaus *et al*. 2009 [25]	Yes	Computer-generated	Unclear	None	Unclear	2
Witt *et al*. 2008 [26]	Yes	Unclear	Unclear	None	Unclear	1

**Table 3:** **The assessment risk of bias**

**Study**	**Sequence generation**	**Allocation concealment**	**Blinding of participants, personnel and outcome assessors**	**Incomplete outcome data**	**Selective outcome reporting**	**Other bias**	**Overall risk of bias**
Liet *et al*. 2006 [14]	Low	Low	Low	Low	Low	Low	Low
Standl *et al.* 2008 [15]	Low	Low	Unclear	Low	Low	Low	Unclear
Akech *et al*. 2010 [16]	Low	Low	Unclear	Low	Low	Low	Unclear
Van Der Linden *et al*. 2013 [17]	Low	Low	Low	Low	Low	Low	Low
Liet *et al*. 2003 [18]	Unclear	Unclear	Low	Low	Low	Low	Unclear
Hanart *et al*. 2009 [19]	Low	Low	Low	Low	Low	Low	Low
Akkucuk *et al*. 2013 [20]	Unclear	Unclear	Unclear	Low	Low	Low	Unclear
Chong Sung *et al*. 2006 [21]	Unclear	Unclear	Unclear	Low	Low	Low	Unclear
Haas *et al.* 2007 [22]	Unclear	Unclear	Unclear	Low	Low	Low	Unclear
Wills *et al*. 2005 [23]	Low	Low	Low	Low	Low	Low	Low
Sahoo *et al*. 2007 [24]	Unclear	Unclear	Unclear	Low	Low	Low	Unclear
Osthaus *et al*. 2009 [25]	Low	Unclear	Unclear	Low	Low	Low	Unclear
Witt *et al*. 2008 [26]	Unclear	Unclear	Low	Low	Low	Low	Unclear

### Overall mortality

**Figure 2:**
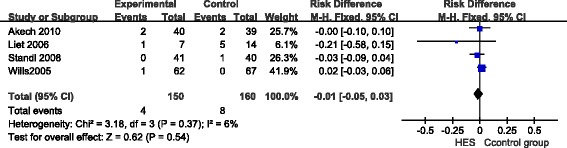
**The forest plot of pooled risk ratio for overall mortality.** CI, confidence interval; HES, hydroxyethyl starch; M-H, Mantel-Haenszel.

In total, four RCTs reported the overall mortality in 310 pediatric patients, including four deaths in 150 children of the HES group and eight deaths in 160 children of the other fluid groups. There was no significant difference in mortality between the HES group and the other fluid groups (RR = -0.01; 95%CI: -0.05 to 0.03; *P* = 0.54; *I*^2^ = 6%) (Figure 2). Funnel plots showed no publication bias (Additional file 2: Figure S1).

### Renal function

**Figure 3:**
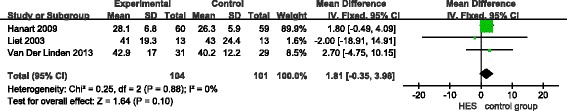
**Forest plot of pooled risk ratio for the effect on renal function (** ***I*** ^***2***^ **-test).** CI, confidence interval; Cr, creatinine; HES, hydroxyethyl starch; IV, inverse variance.

**Figure 4:**
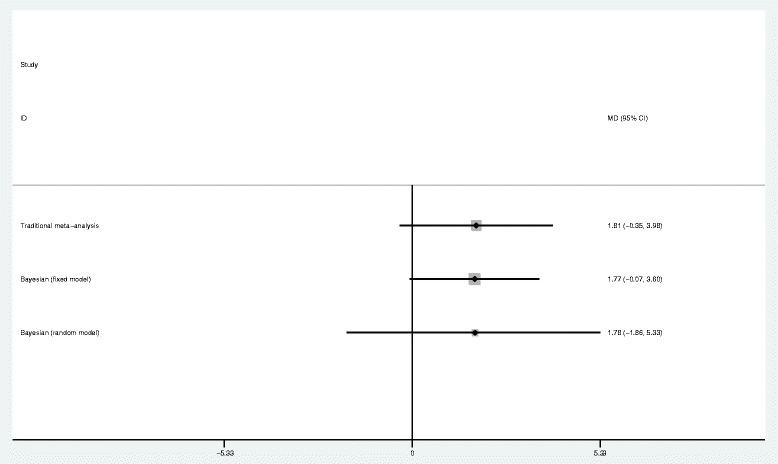
**Forest plot of pooled risk ratio for the effect on renal function (Bayesian analysis with fixed and random effects models developed by Dias** [9]**).** CI, confidence interval; Cr, creatinine; HES, hydroxyethyl starch.

Three RCTs reported Cr change in 205 pediatric patients, including 104 in the HES group and 101 in the control group. The results of statistical analysis showed no significant difference in Cr between the HES group and the other fluid groups (MD = 1.81; 95%CI: -0.35 to 3.98; *P* = 0.10; *I*^2^ = 0%) (Figure 3). Funnel plots showed no publication bias (Additional file 3: Figure S2). Bayesian analysis was performed on Cr using the fixed and random effects models developed by Dias *et al*. [9]. The results showed no significant difference between the two groups (fixed effect model MD = 1.77; 95%CI: -0.07 to 3.6; random effects model MD = 1.78; 95%CI: -1.86 to 5.33) (Figure 4).

### Bleeding and coagulation function

**Figure 5:**
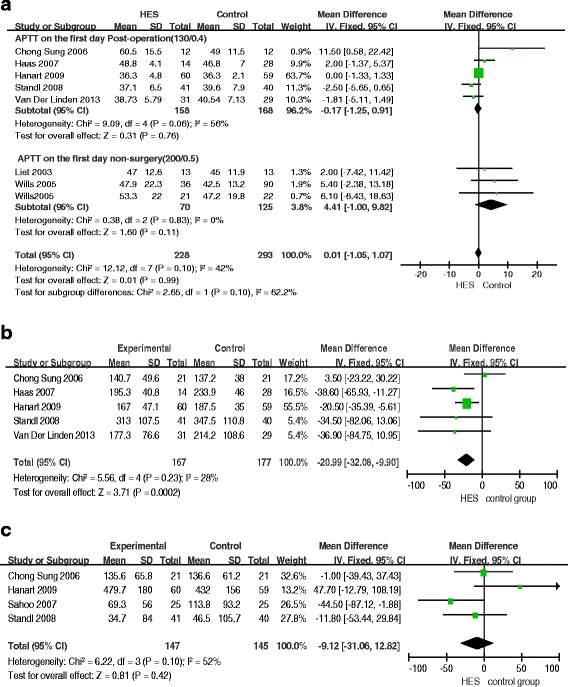
**Forest plots of pooled estimates for the effect on coagulation and bleeding. a)** activated partial thromboplastin time (APTT). **b)** Platelet count. **c)** Blood loss. CI, confidence interval; HES, hydroxyethyl starch; IV, inverse variance.

Seven RCTs reported changes in coagulation function within 24 hours, including 521 pediatric patients (228 in the HES group and 293 in the other fluid groups). We performed subgroup analysis on surgery (five RCTs using 130 kD HES) and non-surgery (two RCTs using 200 kD HES), or different molecular-weight HES. The result showed no significant difference in activated partial thromboplastin time (APTT) between the HES group and the other fluid groups (MD = 0.01; 95%CI: -1.05 to 1.07; *P* = 0.99; *I*^2^ = 42%) (Figure 5a). There was a significant difference in postoperative platelet count (MD = -20.99; 95%CI: -32.08 to -9.90; *P* = 0.0002; *I*^2^ = 28%) (Figure 5b). Four RCTs reported blood loss on the first day post-operation. The result of statistical analysis showed no significant difference in blood loss between the HES group and the other fluid groups (MD = -9.12; 95%CI: -31.06 to 12.82; *P* = 0.42; *I*^2^ = 52%) (Figure 5c). Funnel plots showed no publication bias (Additional file 4: Figure S3a, b, c).

### Hemodynamics

**Figure 6:**
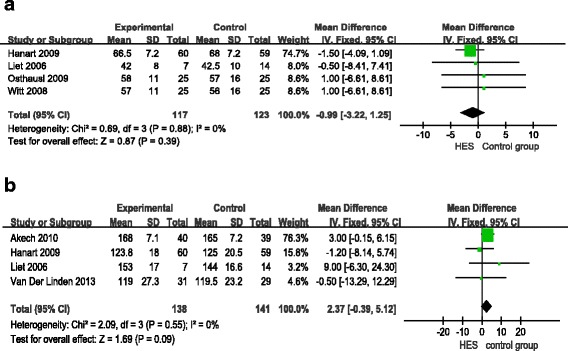
**Forest plots of pooled estimates for the effect on hemodynamics. a)** mean arterial pressure (MAP). **b)** heart rate (HR). CI, confidence interval; HES, hydroxyethyl starch; IV, inverse variance.

Four RCTs reported changes in mean arterial pressure (MAP) and heart rate (HR) on the first day post-operation. The results of statistical analysis showed no significant difference in MAP and HR between the HES group and the other fluid groups (MAP: MD = -0.99; 95%CI: -3.22 to 1.25; *P* = 0.39; *I*^2^ = 0%; HR: MD = 2.37; 95%CI:-0.39 to 5.12; *P* = 0.09; *I*^2^ = 0%) (Figure 6a, b). Funnel plots showed no publication bias. Three RCTs reported the amount of fluid replacement on the first day post-operation. There was significant heterogeneity within 24 hours of surgery (*I*^2^ = 71%). Given multiple factors that may affect the amount of fluid replacement, we only did descriptive analysis without meta-analysis.

### Length of hospital and ICU stay

**Figure 7:**
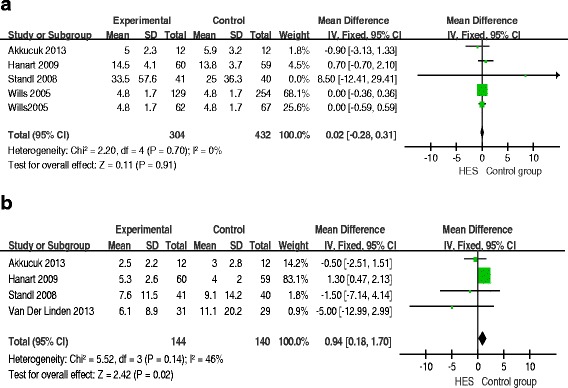
**Forest plots of pooled estimates for the effect on the length of hospital stay and ICU. a)** the length of hospital stay. **b)** the length of ICU stay. CI, confidence interval; HES, hydroxyethyl starch; IV, inverse variance.

Four RCTs reported the length of hospital stay. The results showed no significant difference in the length of hospital stay between the HES group and the other groups (MD = 0.02; 95%CI: -0.28 to 0.31; *P* = 0.91; *I*^2^ = 0%) (Figure 7a). The length of ICU stay in the HES group was longer than that in the other groups (MD = 0.94; 95%CI: 0.18 to1.70; *P* = 0.02; *I*^2^ = 46%) (Figure 7b). Funnel plots showed no publication bias.

## Discussion

The results of this meta-analysis showed that HES significantly decreased the platelet count and increased the length of ICU stay, and also might have had an adverse effect on renal function. Therefore, it should not be recommended for use in pediatric patients before its safe use in children is confirmed by more high-quality RCTs.

HES might have effects on the mortality of pediatric patients. However, this meta-analysis did not show that HES significantly decreased the mortality of pediatric patients as compared with other fluids, which is consistent with the conclusion made by previous studies in pediatric patients [16,23,27-29]. More findings suggest that HES might have adverse effects on the mortality of adult patients. Sedrakyan *et al*. [30] reported that the use of HES was linked with a poorer survival as compared with albumin. Trowbridge *et al*. [31] also reported that elimination of HES was associated with a 67% decrease in the relative odds of death in patients undergoing cardiopulmonary bypass surgery. There still needs more high quality RCTs and studies to confirm the effect of HES on mortality of pediatric patients in future.

This meta-analysis showed HES might have an adverse effect on renal function, which trend was much stronger. Most studies in adult patients also showed that HES had an adverse effect on renal function. This meta-analysis showed that HES did not decrease the Cr level in pediatric patients as compared with other fluids. Considering the wide confidence intervals of the result on Cr and small sample size in the meta-analysis, we cannot conclude that HES is safe in pediatric patients. We used Bayesian analysis to test the possible significant difference. Although compared with previous meta-analysis, Bayesian analysis of the fixed model indicated that the trend was much stronger; the results also showed that there was no significant difference between the two groups. Knowing that many studies reported an association of HES use with an increased incidence of acute kidney injury in adult patients [32-34], it is not unwise to suggest that HES might have adverse effects on renal function and should not be used in pediatric patients with abnormal renal function.

This meta-analysis showed that HES significantly decreased the blood platelet count and might have an adverse effect on the coagulation system in pediatric patients as compared with other fluids, although it did not decrease postoperative blood loss in pediatric patients. Most studies [15,21,25,35] reported that there was no significant change in prothrombin time (PT) and APTT after HES administration in pediatric patients. However, Haas *et al*. [22] reported that HES prolonged APTT and the blood clotting time and significantly increased the hardness of blood clots. Miller BE *et al*. [36] also reported that the increased blood loss after cardiopulmonary bypass in pediatric patients may be associated with the use of HES. Coagulation dysfunction is a common cause of excessive bleeding during and after cardiac surgery. Coagulation dysfunction occurring after cardiopulmonary bypass may be partly caused by platelet loss [37,38]. Thus, platelet loss is a very important adverse effect on heart surgery. These hemostatic concerns about HES have been further substantiated by a meta-analysis of children and adults receiving HES during cardiac surgery, which showed increased blood loss in the patients receiving HES compared with albumin [39]. The present study showed that HES might have an unfavorable effect on the coagulation system in pediatric patients, especially those who underwent heart surgery.

This meta-analysis showed that HES significantly increased the length of ICU stay. Many studies [40-42] reported the same result on the length of hospital stay between the HES group and other solution groups, but we found no related report on the length of ICU stay in adult patients. Given the limited number of studies enrolled in this meta-analysis, more clinical studies with larger sample sizes are needed to confirm the association between HES use and the length of ICU stay.

There are several limitations in this meta-analysis. First, the analysis is based on only 13 RCTs and some of them had a relatively small sample size of pediatric patients. Second, the control groups in these studies used multiple different fluids including fresh frozen plasma, dextran, albumin, gelatin and crystalloids, and, therefore, it is difficult to make a subgroup analysis according to the different fluids. Finally, although there is no heterogeneity between the included studies, patient characteristics including age of the enrolled children and other baseline data are different, which may affect the accuracy of the meta-analysis results.

## Conclusions

Volume expansion with 6% HES significantly decreased the platelet count and increased the length of ICU stay, and also might have an adverse effect on renal function. Therefore, HES is not recommended for use in pediatric patients before more studies confirm these results.

## Key messages

- HES decreased the platelet count and increased the length of ICU stay in pediatric patients, and might have adverse effects on coagulation function.
- HES might have an adverse effect on renal function.
- HES is not recommended for use in pediatric patients before more studies confirm these results.

## Additional files

Additional file 1:
**Search strategy.**
Additional file 2: Figure S1. The funnel plot of overall mortality. Additional file 3: Figure S2. The funnel plot of the effect on renal function. SE,standard error; MD,mean difference. Additional file 4: Figure S3. The funnel plot of the effect on coagulation and bleeding. a) Activated partial thromboplastin time (APTT). b) Platelet count. c) Blood loss. SE,standard error; MD,mean difference.

## Abbreviations

APTT: activated partial thromboplastin time
CI: confidence interval
HES: hydroxyethyl starch
HR: heart rate
ICU: intensive care unit
MAP: mean arterial pressure
MD: mean difference
RCT: randomized controlled trial
RR: relative risk
