# Supplementary material for: Safety evaluation on low-molecular-weight hydroxyethyl starch for volume expansion therapy in pediatric patients: a meta-analysis of randomized controlled trials
Source: Crit Care. 2015 Mar 10;19(1):79. doi: 10.1186/s13054-015-0815-y (PMC4391127; doi:10.1186/s13054-015-0815-y)
Supplement: Additional file 1: — Search strategy. [file 13054_2015_815_MOESM1_ESM.pdf]

Appendix. Search strategy

A, Cochrane Library Issue 1, 2014

#1. MeSH descriptor "Infant,Newborn", Child, "Child,Preschool", Adolescent explode all trees
#2. newborn OR infant OR child* OR 'preschool child*' OR 'school child*' OR adolescent OR juvenile OR neonate OR pediatric
#3. (#1 OR #2)
#4. MeSH descriptor Hydroxyethyl Starch Derivatives explode all trees
#5. "hydroxyethyl* starch*" OR hes* or starch* OR tetrastarch OR tetraspan OR pentastarch OR voluven or hetastarch* OR "haes steril" OR venofundin OR Elohes OR hextend OR "Hydroxyethyl Starch Derivatives" OR Hemohes OR Hespan OR Plasmasteril OR Hydroxyethyl Starch 130-0.4
#6. (#4 OR #5)
#7. (#3 AND #6)

B, MEDLINE (Pubmed) 1980 to January, 2014

((((((((((((((((("Hydroxyethyl Starch Derivatives"[Mesh]) OR hydroxyethyl starch) OR Hydroxyethylated Starches) OR 2-Hydroxyethyl Starches) OR Derivatives, Hydroxyethyl Starch) OR Starches, Hydroxyethylated) OR HAES-steril) OR Fresenius Brand of Pentastarch) OR Hemohes) OR Braun Brand of Pentastarch) OR Hespan) OR Plasmasteril) OR Hydroxyethyl Starch 130-0.4) OR Pentafraction) OR Pentaspan) OR Pentastarch) OR Hetastarch)) AND (((((((("Child"[Mesh]) OR child) OR juvenile) OR children) OR infant) OR neonate) OR newborn) OR pediatric)) AND ((((((((((#42) OR randomised controlled trial[Publication Type]) OR controlled clinical trial[Publication Type]) OR randomised[Title/Abstract]) OR placebo[Title/Abstract]) OR drug therapy[MeSH Subheading]) OR randomly[Title/Abstract]) OR trial[Title/Abstract]) OR groups[Title/Abstract]) NOT ((animals[MeSH Terms]) NOT humans[MeSH Terms]))

C, Embase(OvidSP) 1974 to January, 2014

1. (Newborn or infant or child* or 'preschool child*' or 'school child*' or adolescent or juvenile or neonate or pediatric).af.
2. exp newborn/ or exp juvenile/ or exp newborn/ or exp infant/ or exp preschool child/ or exp school child/ or exp adolescent/
3. 1 or 2
4. ('hydroxyethyl* starch*' or hes* or starch* or tetrastarch or tetraspan or pentastarch or voluven or hetastarch* or 'haes steril' or venofundin or Elohes or hextend or 'Hydroxyethyl Starch Derivatives' or Hemohes or Hespan or Plasmasteril or Hydroxyethyl Starch 130-0.4).af.
5. exp hetastarch/
6. 4 or 5
7. 3 and 6
8. (random* OR factorial* OR crossover* OR placebo*).af.

9. exp crossover-procedure/ or exp double-blind procedure/ or exp randomised controlled trial/ or single-blind procedure/
10. 8 or 9
11. 7 and 10
