# Supplementary figures and images for: Safety evaluation on low-molecular-weight hydroxyethyl starch for volume expansion therapy in pediatric patients: a meta-analysis of randomized controlled trials
Source: Crit Care. 2015 Mar 10;19(1):79. doi: 10.1186/s13054-015-0815-y (PMC4391127; doi:10.1186/s13054-015-0815-y)

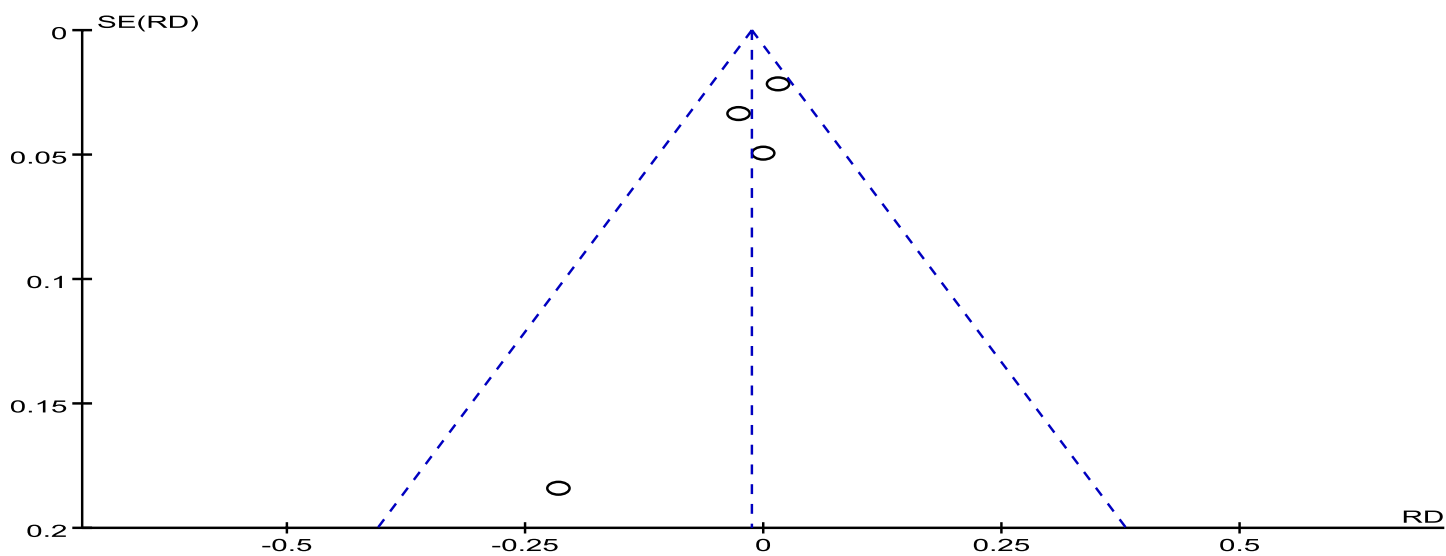

Supplement: Additional file 2: Figure S1. — The funnel plot of overall mortality. [file 13054_2015_815_MOESM2_ESM.pdf]

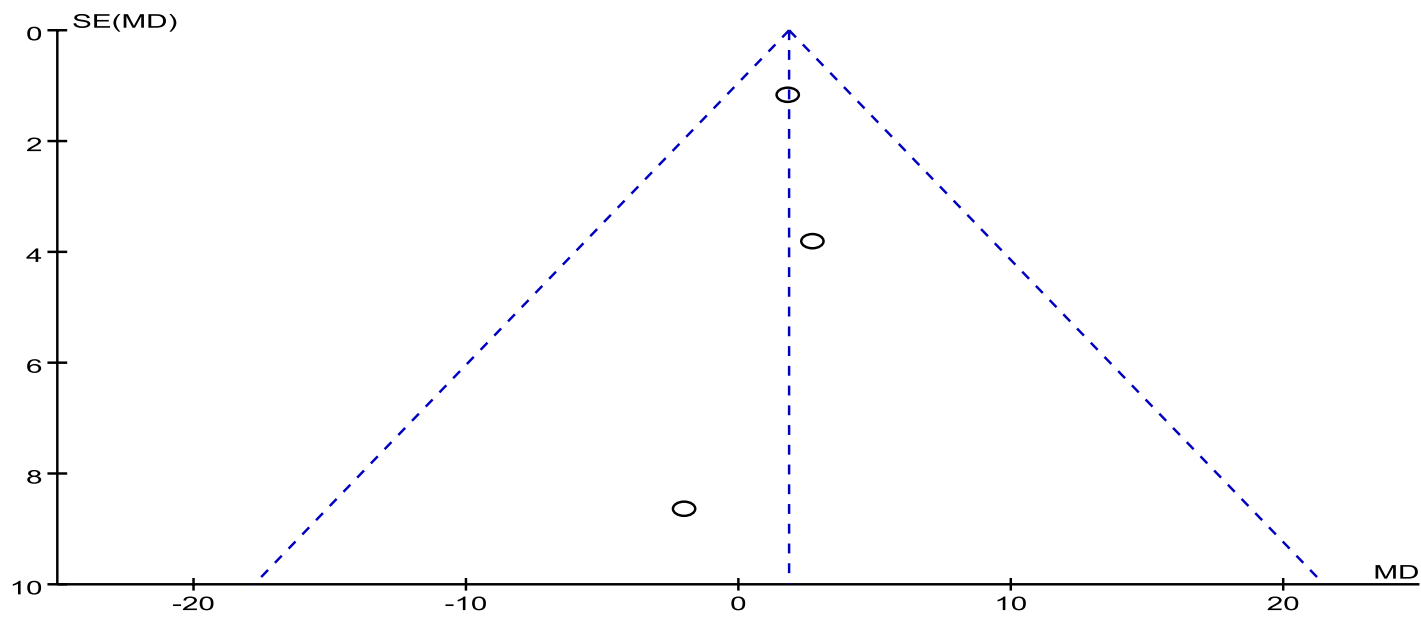

Supplement: Additional file 3: Figure S2. — The funnel plot of the effect on renal function. SE,standard error; MD,mean difference. [file 13054_2015_815_MOESM3_ESM.pdf]

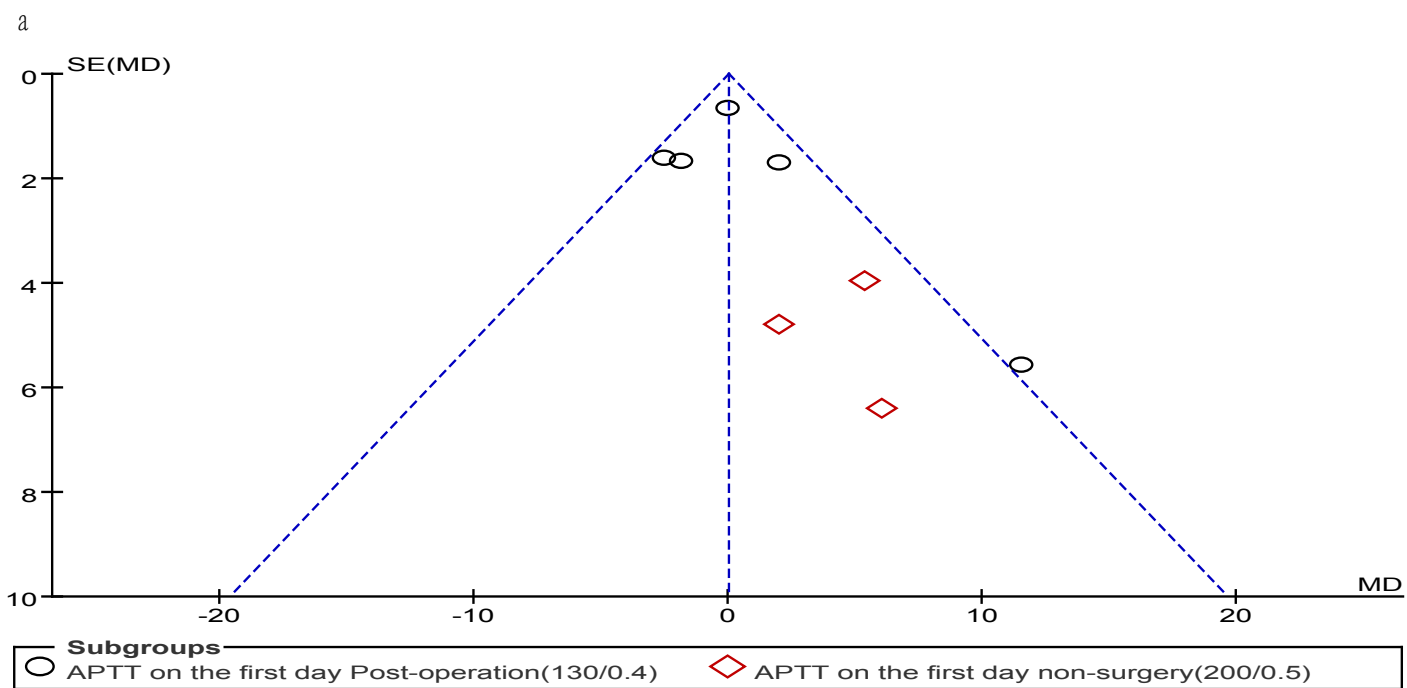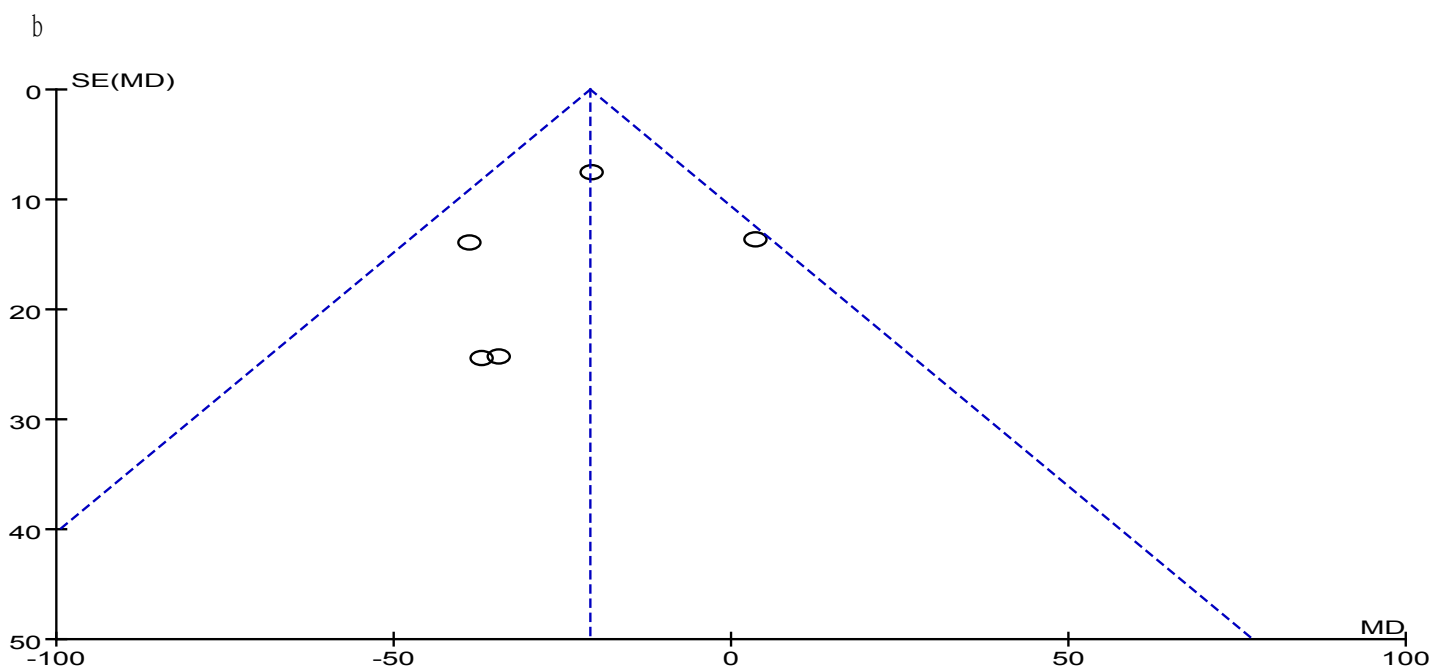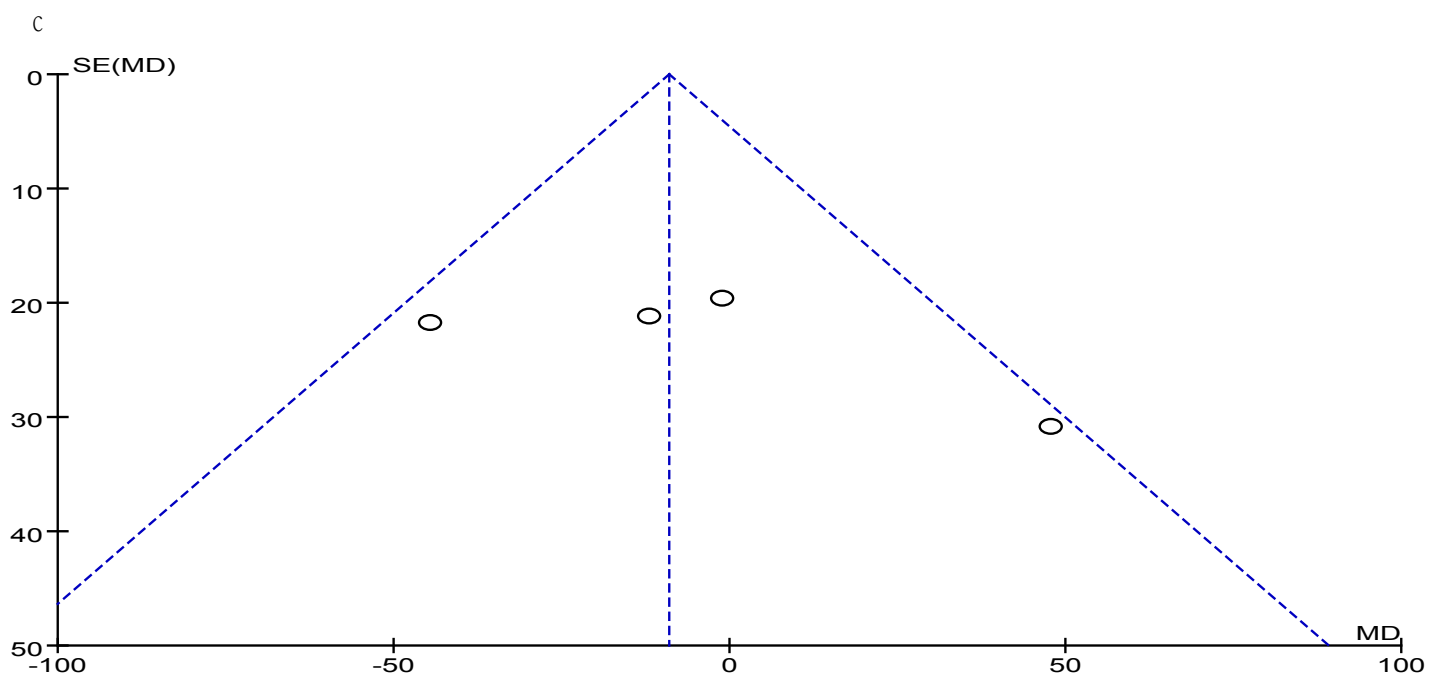

Supplement: Additional file 4: Figure S3. — The funnel plot of the effect on coagulation and bleeding. a) Activated partial thromboplastin time (APTT). b) Platelet count. c) Blood loss. SE,standard error; MD,mean difference. [file 13054_2015_815_MOESM4_ESM.pdf]
